# Reaction Kinetics and Mass Transfer Synergistically Enhanced Electrodes for High-Performance Zinc–Bromine Flow Batteries

**DOI:** 10.1021/acsami.4c22329

**Published:** 2025-04-18

**Authors:** Jiayi Li, Zeyu Xu, Maochun Wu

**Affiliations:** Department of Mechanical Engineering, The Hong Kong Polytechnic University, Hung Hom, Kowloon 999077, Hong Kong SAR, China

**Keywords:** zinc−bromine flow batteries, zinc dendrite, phase field simulation, multiscale electrode, reaction kinetics

## Abstract

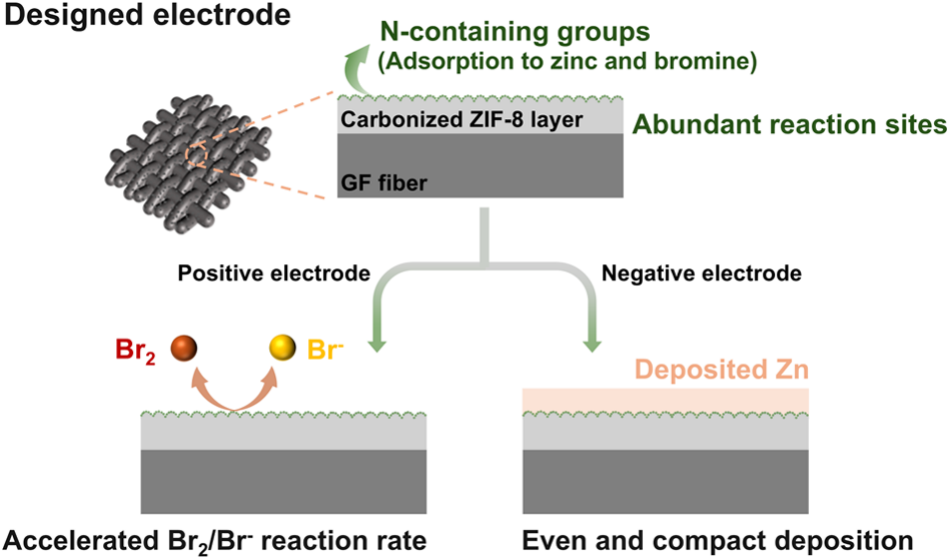

Zinc–bromine flow batteries (ZBFBs) hold great promise for grid-scale energy storage owing to their high theoretical energy density and cost-effectiveness. However, conventional ZBFBs suffer from inhomogeneous zinc deposition and sluggish Br_2_/Br^–^ redox kinetics, resulting in a short cycle life and low power density. Herein, a multiscale porous electrode with abundant nitrogen-containing functional groups is developed by growing zeolitic imidazolate framework-8 in situ on graphite felts, followed by a facile carbonization process to simultaneously tackle both the challenges. Theoretical and experimental results reveal that nitrogen-containing functional groups exhibit a high adsorption energy toward zinc atoms, while the microstructures promote pore-level mass transport, thereby resulting in compact and uniform zinc deposition. In the meantime, the electrode boosts the Br_2_/Br^–^ reaction kinetics due to its high catalytic activity and large surface area. As a result, the ZBFBs equipped with optimized electrodes at both negative and positive sides can operate at an ultrahigh current density of 250 mA cm^–2^ while maintaining an energy efficiency of 68.0%, far surpassing that with pristine graphite felts (50.7%). Remarkably, the battery exhibits excellent cycling stability over 2000 cycles without obvious decay. This study provides a simple yet effective method for developing high-performance electrodes to tackle the critical challenges in ZBFBs, thereby promoting the commercialization of this promising energy storage technology.

## 1 Introduction

Shifting from fossil fuels to renewable energy sources, particularly solar and wind, is essential to achieving carbon neutrality.^1,2^ Nevertheless, the inherent fluctuating nature of these renewables requires large-scale energy storage to maintain grid stability.^3,4^ Of various technologies, aqueous flow batteries have garnered much attention owing to their distinct attributes such as inherent safety, site flexibility, and exceptional scalability.^5−7^ In particular, ZBFBs stand out as one of the most attractive candidates because of their high theoretical specific energy (440 Wh kg^–1^), high cell potential (∼1.85 V), and the use of inexpensive and widely available materials.^8,9^ Despite these advantages, the practical application of ZBFBs is hindered by their limited operating current density and short cycle life.^10,11^ The former primarily results from sluggish Br_2_/Br^–^ reaction kinetics on the positive electrode, while the latter is due to dendrite formation and “dead Zn” associated with shedding issues on the negative side, which considerably jeopardizes the efficiency and longevity of ZBFBs.^12−15^

Electrodes, which offer not only active sites for electrochemical reactions but also ion/mass transport pathways, play a key role in determining the performances of ZBFBs.^16,17^ Currently, carbon-based materials, especially graphite felts (GFs), are common electrode materials for ZBFBs owing to their superior electrical conductivity, high stability, high porosity, and low cost.^18−20^ However, conventional GFs only offer a limited active surface for electrochemical reactions due to the smooth, highly graphitized fiber surface. When used as negative electrodes, the smooth fiber surface results in few Zn deposition sites and insufficient adhesion to the Zn deposits. Consequently, the deposited Zn tends to grow into dendrites and easily falls off with the flow of electrolyte during charging and discharging processes, greatly reducing Coulombic efficiencies (CEs) and shortening the cycle life of ZBFBs.^21^ When applied as positive electrodes, ZBFBs suffer from large polarizations, leading to low energy efficiencies (EEs) and limiting operating current density.^4^ In the past, tremendous efforts have been devoted to tackling these challenges by modifying the electrode surface properties. For negative electrodes, Sun et al. and Lee et al. demonstrated that the single-vacancy carbon defect presents high bonding energy with Zn and can prevent surface diffusion of Zn, thus effectively suppressing Zn dendrite growth.^22,23^ Lu et al. reported that incorporating N-rich defects into electrodes significantly increases the Zn adsorption capacity while creating more nucleation sites for Zn deposition.^21^ Additionally, Yin et al. developed a tin (Sn)-deposited carbon felt to achieve uniform Zn plating/stripping thanks to the strong Zn–Sn interaction and enhanced hydrogen evolution overpotential of Sn.^24^ As for the positive side, major efforts were mainly focused on improving the electrochemical activity of conventional GFs for the Br_2_/Br^–^ reaction by heteroatom (e.g., oxygen^20^ and nitrogen^25^) doping and application of electrocatalysts (e.g., platinum,^26^ titanium nitride,^12^ and carbon nanotube^27^). Despite the tremendous progress, the performance of ZBFBs still remains inferior, particularly when compared to that of its all-vanadium counterpart. This is because most previous studies mainly focused on solving only one specific issue, rendering the overall performance unsatisfactory. Therefore, it remains a great challenge to develop electrodes that can simultaneously address Zn dendrite formation and sluggish Br_2_/Br^–^ reaction kinetics.

Herein, a nitrogen-doped, multiscale porous electrode was designed and fabricated to simultaneously address both challenges in ZBFBs. Density functional theory (DFT) calculations reveal that the N-containing functional groups provide high affinity toward Zn atoms and bromine molecules, thus promoting compact zinc deposition and enhancing Br_2_/Br^–^ conversion rates. Phase field simulation further unravels that the microstructured surface effectively promotes dendrite-free deposition due to the enhanced interfacial mass transport. It is demonstrated that the ZBFBs equipped with the newly designed positive and negative electrodes are capable of operating at an ultrahigh current density of 250 mA cm^–2^ with an EE of 68.0% and show no obvious degradation for 2000 cycles at 100 mA cm^–2^, whereas the battery with pristine graphite felt (PGF) electrodes delivers an EE of as low as 50.7% at 250 mA cm^–2^ and suffers from fluctuation only after about 50 cycles at 100 mA cm^–2^.

## 2 Results and Discussion

**Figure 1:**
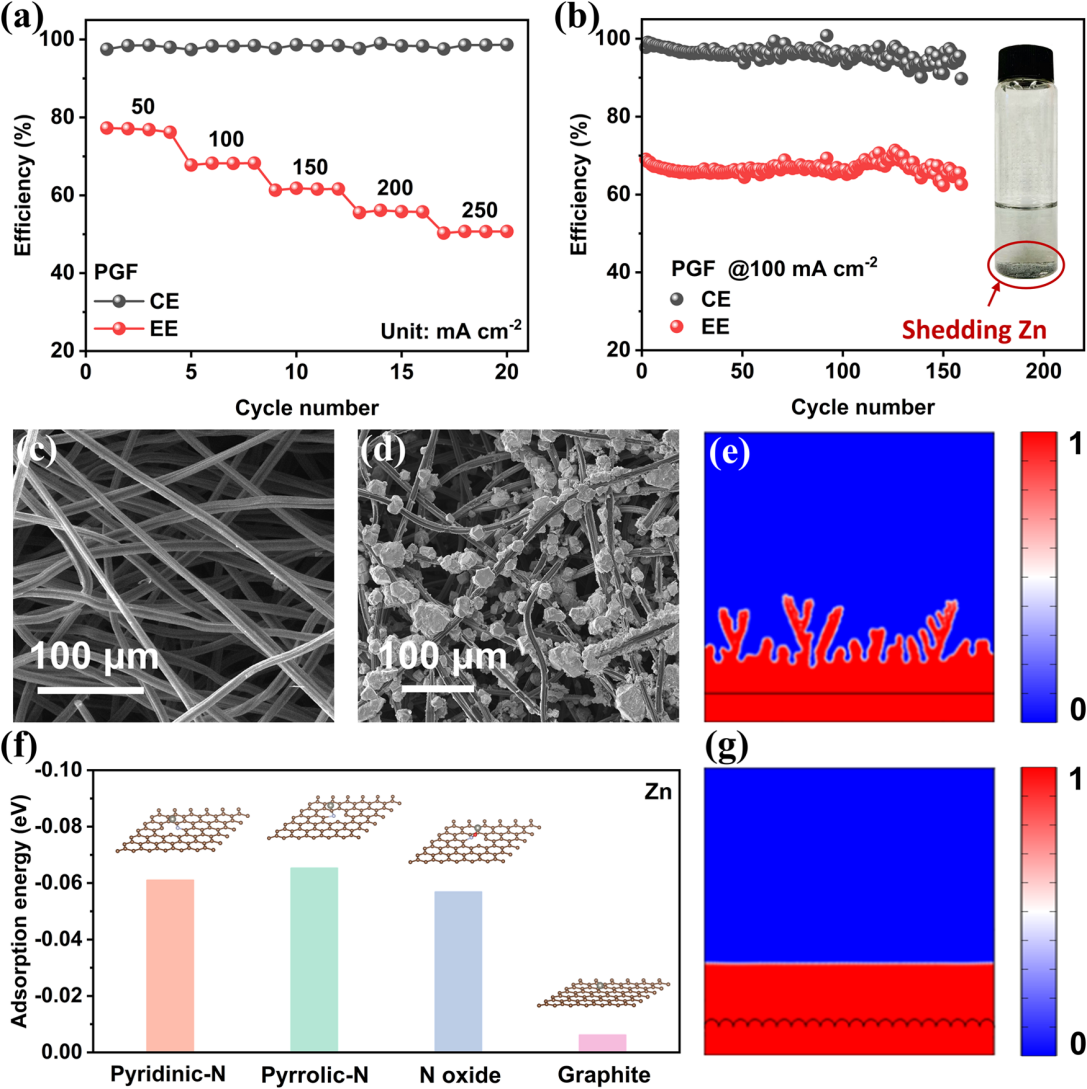
(a) CE and EE of ZBFB using PGF as both positive and negative electrodes at different current densities. (b) Zn shedding in the electrolyte tank and cycling performance of ZBFB with PGF electrodes at 100 mA cm^–2^. SEM image of (c) PGF and (d) Zn deposited on the PGF negative electrode on the membrane side after 12 min of charge at 100 mA cm^–2^. (e) Phase field simulation of Zn deposition on the PGF electrode surface. (f) Adsorption energy of zinc atoms on different electrode surfaces. (g) Phase field simulation of Zn deposition on the designed electrode surface.

Figure 1a shows the rate performance of ZBFB assembled with PGF electrodes. It is found that even at a low current density of 50 mA cm^–2^, the EE is only about 76.2% and continues dropping to 50.7% when the current density reaches 250 mA cm^–2^, indicating the high polarization resulting primarily from low electrochemical activity of PGFs. Cycling test in Figure 1b further reveals that the CE of ZBFB decreases significantly and then becomes unstable after around 50 cycles at 100 mA cm^–2^. Meanwhile, shedding Zn was observed in both the negative electrolyte tank and tube. This is due to the nonuniform Zn deposition and some Zn may be flushed away by the flowing electrolyte due to the insufficient binding of Zn and electrode, which is consistent with previous works.^28−30^ To better understand the origin of shedding Zn, surface morphologies before and after Zn deposition were observed by scanning electron microscopy (SEM). As shown in Figure 1c, PGF exhibits a smooth fiber surface that can provide only limited active sites for Zn deposition. As expected, the deposited Zn on PGF is nonuniform in both size and spatial distribution (Figure 1d) after being charged at 100 mA cm^–2^ for 12 min (corresponding to 20 mAh cm^–2^). It can be presumed that the adhesion between Zn grains and fiber surfaces is weak, which could easily lead to Zn abscission, thus decreasing the CE and cycle stability of ZBFBs. Phase field simulations were then conducted to reveal the underlying mechanism of dendrite formation. As shown in Figure 1e and Video S1, during the electrodeposition process, the electrode–electrolyte interface moves toward the electrolyte and becomes rough. Further growth of these unevenly deposited Zn results in rampant dendrite formation. During the discharge process, these dendrites are prone to break and fall off from the electrode surface during the dissolution process to form “dead zinc”, which is flushed away by the flowing electrolyte, resulting in low CE and poor stability.

To gain insights into the shedding of Zn, DFT calculations were carried out to study the interaction of Zn atoms with the pristine graphite surface (Figure 1f). It was found that the absorption energy is as low as −0.006 eV, indicating the weak adsorption between Zn and pristine electrode, which is a root cause of Zn shedding during battery operation. An effective solution to address this issue is to design a surface that exhibits a high binding energy toward Zn atoms. As heteroatom doping is one of the most promising methods to modulate the surface properties, we calculated the adsorption energies of *N*-containing defects toward Zn atoms, which are −0.061, −0.065, and −0.057 eV for pyridinic-*N*, pyrrolic-*N*, and *N* oxide, respectively, which are much more negative than that on the pristine graphite surface. The lone pair electrons of these *N*-containing groups can coordinate with Zn^2+^, reducing the nucleation energy barrier and promoting uniform nucleation.^31−33^ These results indicate N-doping is a promising way to enhance the adsorption ability to Zn atoms, which is conducive to anchoring Zn and thus promotes compact Zn deposition. Moreover, additional charge carriers introduced by *N*-containing groups can enhance the uniform distribution of electric field on the electrode surface, thereby alleviating local current concentration that may lead to uneven Zn deposition.^34^

In addition to the surface properties that largely determine the nucleation process, uniform ion distribution at the electrode/electrolyte surface is critical to dendrite-free Zn deposition.^35,36^ We conjecture that constructing microprotrusions on the smooth fiber surface will promote mass transfer at the interface, thus suppressing dendrite growth. Phase field simulation was then performed to preliminarily validate this hypothesis. Numerical results in Figure 1g and Video S2 reveal that the protrusions formed during the electrode modification process are indeed beneficial to the flat, uniform, and dendrite-free Zn formation. This is attributed to the fact that the rough surface promotes the uniform distribution and transfer of Zn^2+^ ions at the electrode/electrolyte interface.^37^

**Figure 2:**
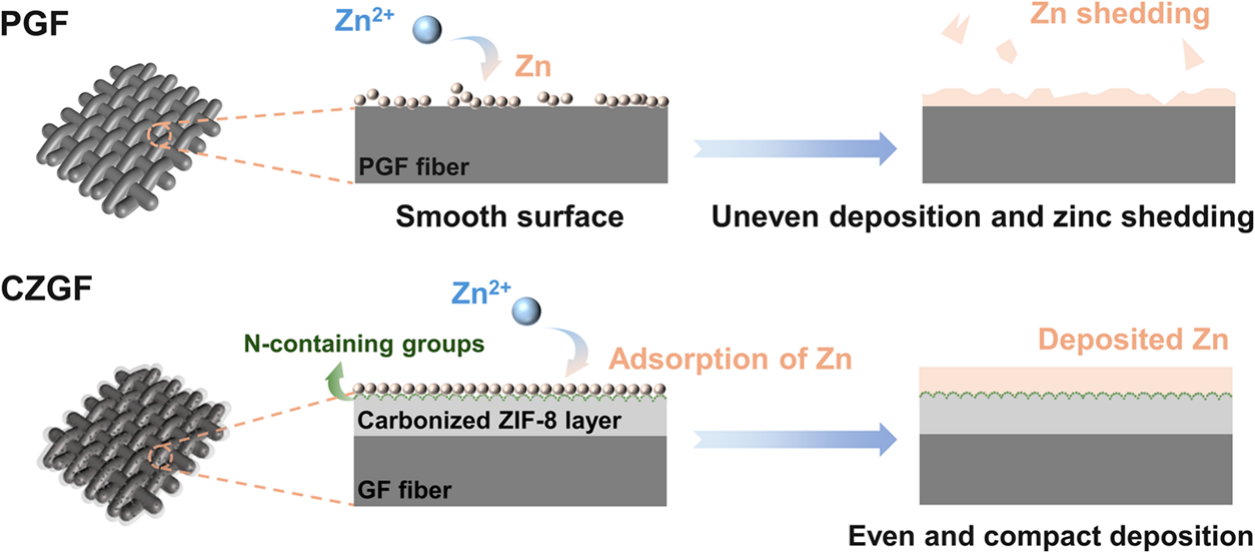
Schematic Illustrations of Zn Deposition on PGF and Designed CZGF Electrodes.

Guided by the above fundamental understanding, a N-doped, multiscale porous electrode was proposed by carbonizing ZIF-8 in situ growth on GFs (CZGFs). As illustrated in Figure 2, when using PGF as a negative electrode, which has fewer and uneven Zn nucleation sites, the Zn deposit is uneven and prone to shedding. By contrast, the designed CZGF electrode with a rough N-containing layer derived from carbonized ZIF-8 not only provides abundant sites with strong adsorption for Zn nucleation but also enhances the interfacial mass transfer of Zn^2+^ ions due to the increased surface flow velocity (Figure S1), thereby achieving uniform and dense Zn deposition. Figure S2 illustrates the synthesis procedure of the CZGFs. First, PGF was immersed in a mixed solution of Zn(NO_3_)_2_·6H_2_O and 2-methylimidazole for in situ growth of ZIF-8 particles. As the carbonization temperature is a key parameter that determines the removal of Zn during pyrolysis and significantly influences structural and compositional properties of the resulting electrodes, the ZIF-8-modified GFs (ZGFs) were then carbonized at 700, 800, 900, and 1000 °C for 5 h under the N_2_ atmosphere to achieve different CZGFs (denoted as CZGF-700, CZGF-800, CZGF-900, and CZGF-1000).

**Figure 3:**
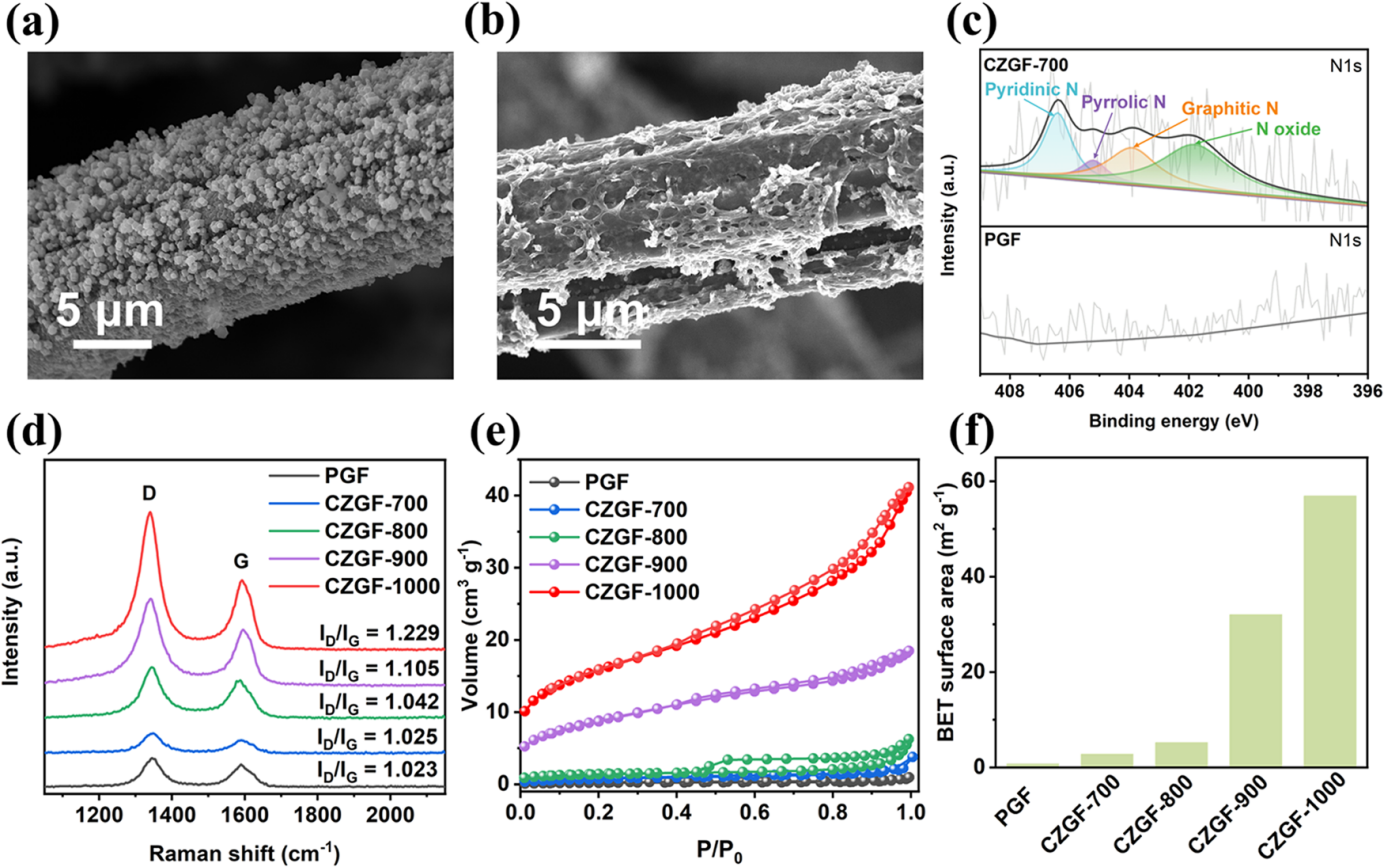
SEM images of (a) ZGF and (b) CZGF-700 electrode surfaces. (c) N 1s XPS spectra of CZGF-700 and PGF. (d) Raman spectra, (e) nitrogen adsorption–desorption isotherms, and (f) BET surface area of different electrodes.

SEM images in Figures 3a and S3 show that the PGF fiber surface is uniformly coated with rhombic polyhedral ZIF-8 particles after soaking in the precursor solution. X-ray diffractometer (XRD) patterns in Figure S4a reveal that the ZGF shows typical diffraction peaks corresponding to the (011), (002), (112), (022), (013), and (222) crystal planes of ZIF-8, confirming successful growth of ZIF-8 on PGF.^38,39^ After carbonization under a N_2_ atmosphere at 700 °C, the ZIF-8 nanoparticles disappear and are transformed into a rough carbon layer coated on the surface of GF fibers (Figure 3b). This is because ZIF-8 particles undergo partial decomposition at 600 °C and are fully transformed to carbon at 700 °C, which is evidenced by the shift in the XRD patterns of CZGF-700 shown in Figure S4b. During this process, the decomposition of ZIF-8 results in the formation of carbon with ZnO, simultaneously facilitating the formation of porous structures.^40^ SEM images of CZGFs obtained under various carbonization temperatures are shown in Figure S5. It is found that as the carbonization temperature increases, the particle size in the carbonized ZIF-8 layer gradually decreases. This may be due to the fact that the higher carbonization temperature will intensify the pyrolysis process, enabling reduction of ZnO and evaporation of Zn to form a more porous and defect-rich structure.^23^ Then, the composition and elemental chemical states of the prepared electrodes were analyzed by X-ray photoelectron spectroscopy (XPS). Full XPS spectra survey (Figures S6 and S7a) reveals that the carbonization of ZIF-8 successfully incorporates *N*-containing functional groups to the electrodes. As shown in Figures 3c and S7b, the N 1s spectrum is deconvoluted to four peaks located at 398.6, 399.8, 401.1, and 403.2 eV, which correspond to pyridinic-*N*, pyrrolic-*N*, graphitic-*N*, and *N* oxide, respectively.^41^ These *N*-containing functional groups are beneficial to increasing the adsorption ability to Zn atoms and thus promote uniform and robust Zn deposits. Raman spectra were also acquired to probe the surface properties of CZGFs. As displayed in Figure 3d, all samples exhibit two typical peaks. One centered at ∼1342 cm^–1^ is the D band that is associated with the disordered carbon or other carbon defects, while the other located at ∼1590 cm^–1^ corresponds to the G band related to graphitic carbon. In general, the amount of defects in the carbon materials was characterized by the intensity ratio of D to G band (*I*_D_/*I*_G_).^42^ The values of *I*_D_/*I*_G_ for PGF, CZGF-700, CZGF-800, CZGF-900, and CZGF-1000 are calculated to be 1.023, 1.025, 1.042, 1.105, and 1.229, respectively. As the temperature increases, nitrogen atoms gradually decompose or volatilize, leaving behind vacancies and edge defects in the carbon lattice.^43^ These defects contribute to an increase in *I*_D_/*I*_G_, leading to a higher *I*_D_/*I*_G_ ratio, despite the overall graphitization trend. Moreover, Zn vaporization above 908 °C creates pores, further amplifying disordered carbon domains and enhancing the surface area of electrodes.^44−46^Figure 3e displays the N_2_ adsorption–desorption isotherms of different electrodes. It is found that the adsorption capacity of the electrode increases with an increasing carbonization temperature. Moreover, the hysteretic loop of the CZGF-1000 electrode can be classified to type IV, which reveals the existence of numerous mesopores and micropores and is further verified by the pore size distribution in Figure S8.^47^Figure 3f depicts that the Brunauer–Emmett–Teller (BET) surface area increases with the carbonization temperature. Thanks to the presence of these meso- and micropores, the specific surface area dramatically increases from 0.72 m^2^ g^–1^ for PGF to 56.91 m^2^ g^–1^ for CZGF-1000, thereby considerably increasing the reaction sites.

**Figure 4:**
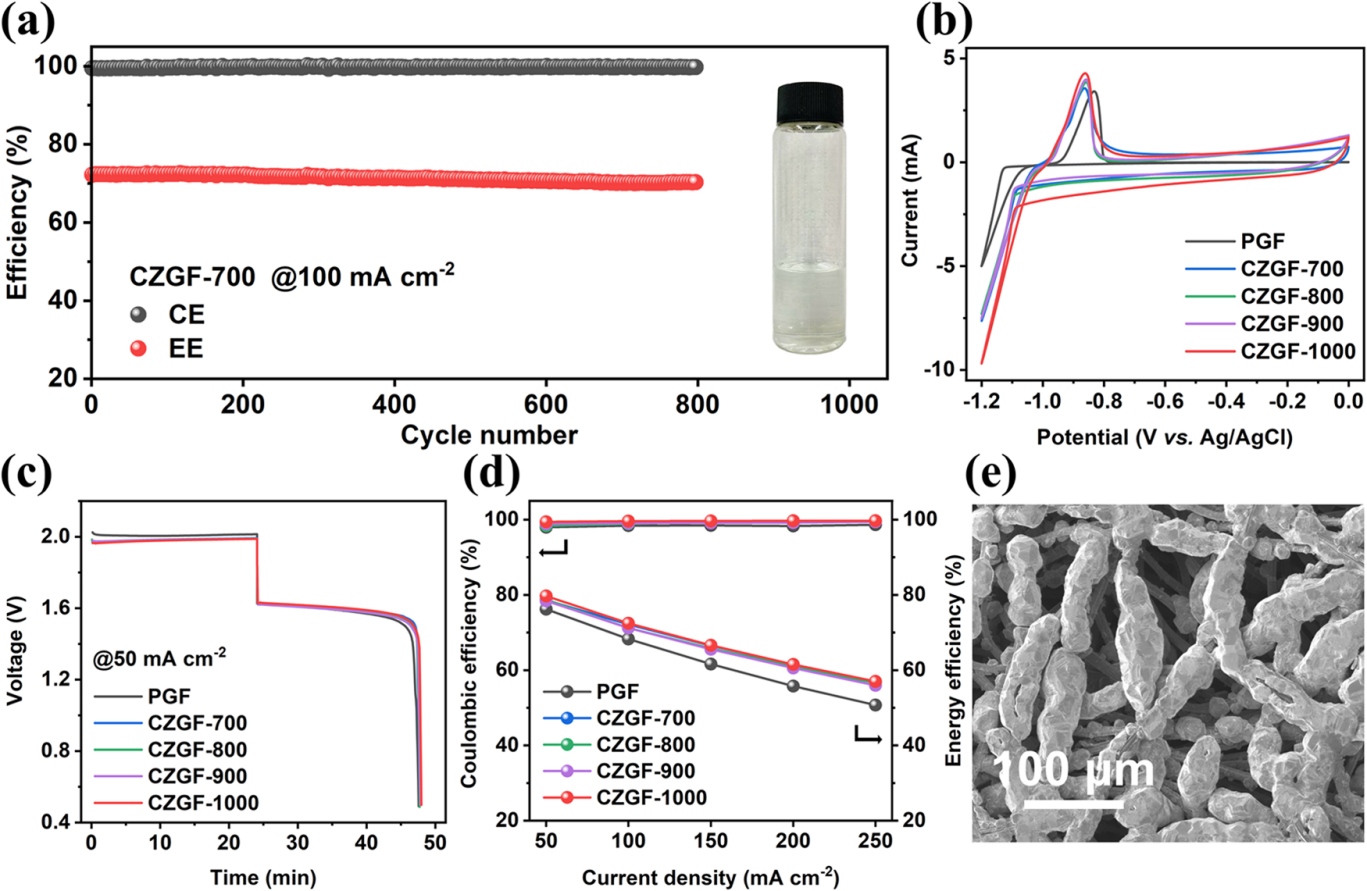
(a) Cycling performance of ZBFB using CZGF-700 and PGF as negative and positive electrodes at 100 mA cm^–2^ and the negative electrolyte after cycling. (b) CV profiles of different samples at a scan rate of 10 mV s^–1^. (c) Charge–discharge profiles of ZBFBs with different CZGF negative electrodes and the same PGF positive electrodes at 50 mA cm^–2^. (d) CE and EE of ZBFBs with various negative electrodes and the same PGF positive electrodes at different current densities. (e) SEM image of Zn deposited on the CZGF-1000 negative electrode on the membrane side after 12 min of charge at 100 mA cm^–2^.

To experimentally validate our hypothesis of the designed electrodes for dendrite-free Zn deposition, a ZBFB was assembled with CZGF-700 as the negative electrode and PGF as the positive electrode and cycled at 100 mA cm^–2^. The results show that the ZBFB can cycle stably for over 800 cycles without obvious degradation, and no detached Zn is observed in the electrolyte tank and tubes (Figure 4a), confirming the effectiveness of our strategy in tackling the critical challenge of Zn electrodes. The cyclic voltammetry (CV) test was then performed to evaluate the Zn deposition/dissolution behavior on different electrodes. As displayed in Figures 4b and S9, the initial Zn deposition potentials on PGF, CZGF-700, CZGF-800, CZGF-900, and CZGF-1000 are −1.146, −1.100, −1.106, −1.104, and −1.103 V, respectively. The difference in the initial deposition overpotential of zinc on different CZGF electrodes is not obvious. However, the initial Zn deposition potential with CZGFs changes positively and the deposition overpotential is reduced by about 43 mV compared to the PGF, demonstrating that a smaller Zn deposition barrier needs to be overcome,^48^ which facilitates the uniform deposition of Zn.^49,50^ All cathodic currents and anodic peak currents of CZGFs are higher than those of PGF. As the carbonization temperature increases, the anodic peak current gradually increases, and CZGF-1000 shows the highest results among all tested electrodes. Moreover, the anodic peak potential of CZGFs shifts negatively compared with PGF, indicating a certain increase in reversibility for the Zn^2+^/Zn reactions. In addition, ZBFBs were also assembled to assess the effect of using modified CZGFs as negative electrodes on the battery performance. The charge–discharge voltage curves of ZBFBs with various CZGF negative electrodes and the same PGF positive electrodes were compared in Figure 4c. It is found that replacing PGF with CZGF as a negative electrode reduces charge voltage plateaus and improves discharge voltage plateaus of ZBFBs. There is no obvious difference in the charge and discharge voltage plateaus of ZBFBs using varied CZGF negative electrodes, indicating the carbonization temperature has no significant effect on the Zn^2+^/Zn reaction kinetics. Figure 4d displays the CEs and EEs of the ZBFBs tested under different current densities. Encouragingly, the batteries installed with the CZGF negative electrodes exhibit high CEs exceeding 99.2%, which is about 1% higher than that with PGF. This is primarily ascribed to the improved Zn deposition. The ZBFB with the CZGF-1000 negative electrode also exhibits exceptional cycling stability at 100 mA cm^–2^ with no obvious performance degradation after about 800 cycles (Figure S10). As shown in Figure 4e, dense, compact Zn electrodeposits are found on CZGFs, which fully cover the fiber surface. Although EEs are also improved when CZGFs are used as negative electrodes, probably due to the slightly enhanced reaction kinetics or increased surface area, the improvement is limited and the EEs are relatively low (∼56.6%) when operated at a high current density of 250 mA cm^–2^. It is inferred that the larger polarization mainly comes from the positive electrode,^51^ which will be addressed in the following section. More remarkably, when deposited at a higher areal capacity of 40 mAh cm^–2^, CZGF-1000 can still maintain a uniform and flat Zn deposition (Figure S11), whereas Zn is unevenly deposited on the surface of PGF. These results successfully confirm that the newly designed CZGF electrodes can effectively induce dendrite-free Zn deposition, thereby greatly extending the cycle life of ZBFBs.

**Figure 5:**
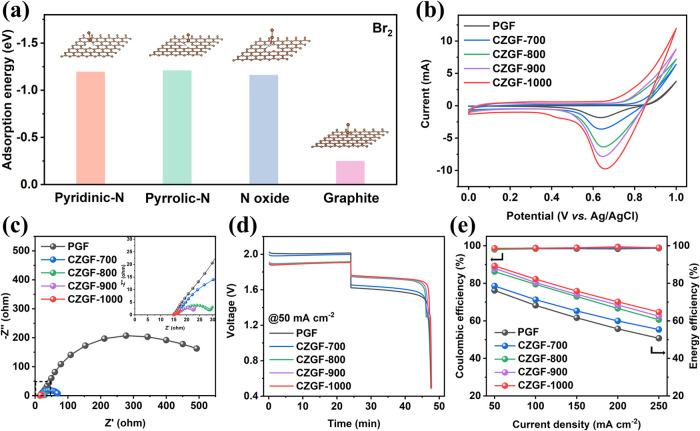
(a) Adsorption energy of bromine on representative surfaces of CZGF and PGF. (b) CV profiles of different electrodes with the potential ranging from 0 to 1 V vs. Ag/AgCl. (c) Nyquist plots of different samples. (d) Voltage–time profiles of ZBFBs with different positive electrodes during the charge–discharge processes at 50 mA cm^–2^. (e) CE and EE of ZBFBs with different positive electrodes when charged and discharged at various current densities.

As mentioned above, the sluggish Br_2_/Br^–^ reaction kinetics is another critical barrier hindering the development of ZBFBs. Inspired by the unique features of *N*-containing functional groups, DFT calculations were performed to examine their adsorption capability toward bromine molecules. As shown in Figure 5a, all *N*-containing groups exhibit lower adsorption energies than the pristine carbon surface due to the fact that these *N*-containing groups contain more electrons and have a strong adsorption capacity for bromine molecules, which are more inclined to gain electrons, resulting in a much faster rate of the Br_2_/Br^–^ reaction.^19^ CV tests were subsequently conducted to evaluate the Br_2_/Br^–^ reaction kinetics using CZGF electrodes. Figure 5b shows that the cathodic peak currents and oxidation currents of CZGFs are higher than those of PGF in the CV curves, suggesting the improved electrode activity. With the carbonization temperature increasing, the cathodic peak current also increases and the highest cathodic peak current is obtained with CZGF-1000 due to its largest active surface area among these CZGFs. Electrochemical impedance spectroscopy (EIS) tests further elucidated the electrochemical processes occurring on different electrodes. Nyquist plots in Figure 5c and the equivalent circuit in Figure S12 reveal that the bulk solution resistances (*R*_s_) with PGF and CZGFs are around 15 Ω, indicating little difference in electrical conductivity for these samples, while the semicircle diameters exhibit a progressive reduction in the order of PGF > CZGF-700 > CZGF-800 > CZGF-900 > CZGF-1000, suggesting the reduced charge transfer resistance (*R*_ct_) with CZGFs. This also confirms that the CZGF-1000 electrode possesses the fastest kinetics toward Br_2_/Br^–^ reactions among all modified CZGFs, consistent with the CV results.

ZBFBs with different positive electrodes and PGF negative electrodes were then assembled to further evaluate their electrochemical performances. As shown in Figure 5d, a sequential reduction in charge voltage plateau in the order of PGF > CZGF-700 > CZGF-800 > CZGF-900 > CZGF-1000 is observed, while the discharge plateau increases in the reverse trend. Accordingly, the ZBFB assembled with the CZGF-1000 positive electrode delivers the highest EE of 89.3% at 50 mA cm^–2^, far outperforming that with PGF electrodes (76.2%). This is primarily ascribed to enhanced reaction kinetics and surface area of CZGF-1000 electrodes. The EE and CEs of ZBFBs with different positive electrodes operating from 50 to 250 mA cm^–2^ are summarized in Figure 5e. The CEs display an increase trend with increasing current density, primarily attributed to reduced bromine crossover, while reduced EEs result from increased polarization under elevated current densities.^52^ The ZBFBs with modified CZGF positive electrodes exhibit higher EEs than that with PGF electrode under all tested current densities, indicating the fast reaction kinetics of the Br_2_/Br^–^ couple. Remarkably, the CZGF-1000 positive electrode enables ZBFB to achieve a high EE of 64.7% even at 250 mA cm^–2^, confirming the excellent rate performance.

**Figure 6:**
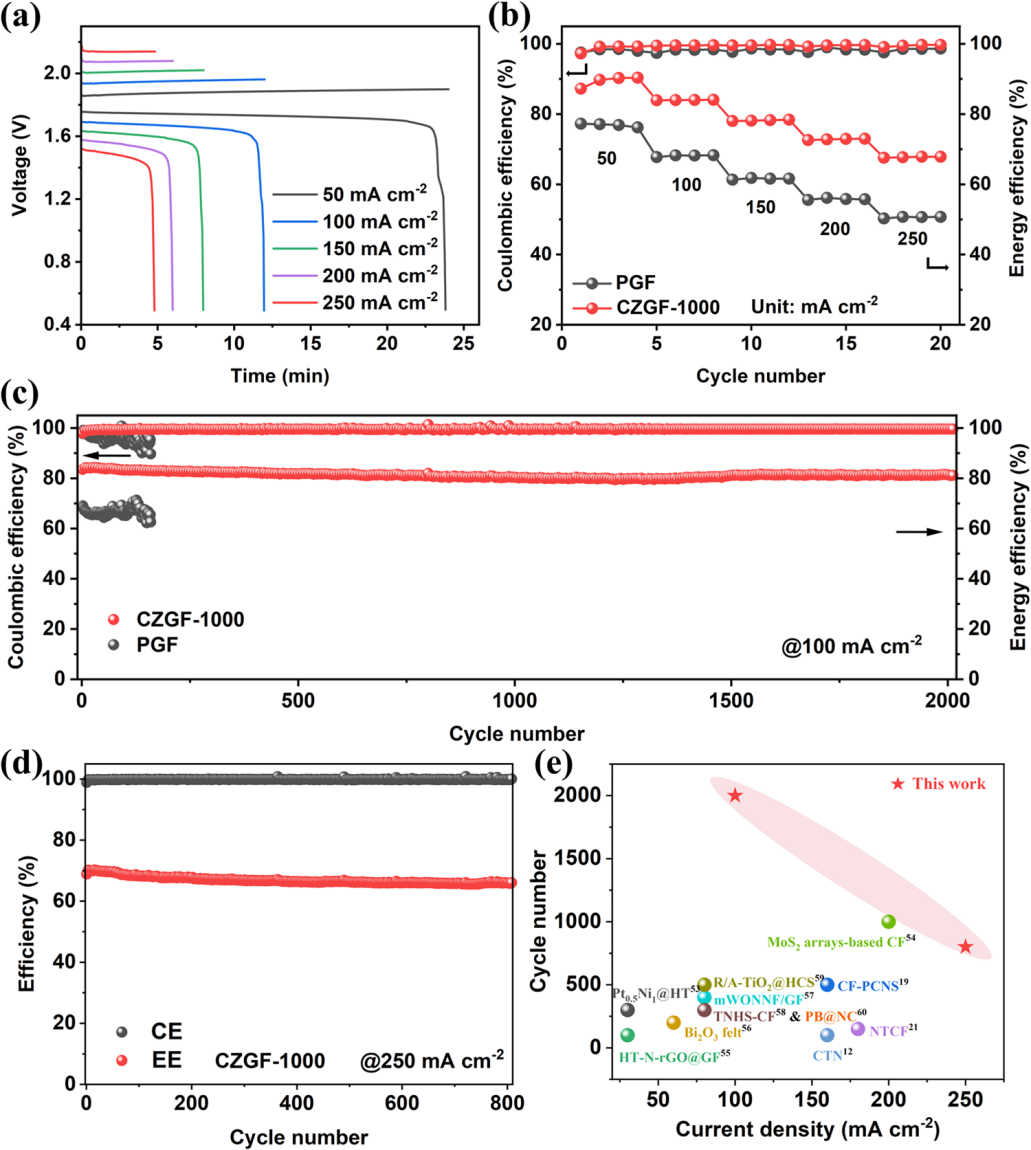
(a) Voltage–time profiles and (b) CE and EE of ZBFBs using CZGF-1000 as both negative and positive electrodes at different current densities. (c) Cycling performances of ZBFBs with both PGF and CZGF-1000 electrodes at 100 mA cm^–2^ and 20 mAh cm^–2^. (d) Cycling performance with both CZGF-1000 electrodes at 250 mA cm^–2^ and 20 mAh cm^–2^. (e) Comparison of operating current density and cycling number of ZBFBs using CZGF-1000 electrodes with recently reported related works.^12,19,21,53−60^

Eventually, the performances of ZBFBs with both positive and negative CZGF-1000 electrodes were evaluated with increasing current densities. The resulting charge–discharge curves presented in Figure 6a show that as the current density increases, the gaps between the charge and discharge voltage increase as a result of the increased polarizations. Additionally, the performances of ZBFBs with both PGF and CZGF-1000 operated at different current densities are presented in Figure S13. It is clearly seen that the gaps between the charging and discharging plateaus of the ZBFB with CZGF-1000 electrodes are smaller than those with PGF, thus leading to a much higher EE. Figure 6b presents the corresponding CEs and EEs of the ZBFBs with both the CZGF-1000 and PGF electrodes. The results show that the CEs of battery using CZGF-1000 electrodes reach 99.7% as a result of dendrite-free Zn deposition, which are higher than that with PGF electrodes. Impressively, ZBFB installed with CZGF-1000 electrodes exhibits an ultrahigh EE of 90% at 50 mA cm^–2^ and can still maintain an EE of 68.0% at 250 mA cm^–2^, far surpassing that equipped with PGF electrodes. To assess the durability of CZGFs, the ZBFBs with CZGF-1000 as both negative and positive electrodes were cycled at different current densities. The results in Figure 6c show that the ZBFB can maintain an EE above 81% over 2000 stable cycles (over 800 h) when operated at 100 mA cm^–2^. Notably, even at 250 mA cm^–2^, the ZBFB still exhibits exceptional stability over 800 cycles (Figure 6d), which exceeds the performance reported in most previous works on ZBFBs shown in Figure 6e and Table S1. In addition, the polarization test was conducted, and the result is shown in Figure S14. It is found that the ZBFB with CZGF-1000 electrodes can deliver a maximum power density of as high as 707.6 mW cm^–2^, far surpassing that with PGFs (432.4 mW cm^–2^), indicating the effectiveness of our strategy in enhancing the power output of ZBFB. These results demonstrate that CZGFs can not only promote dendrite-free Zn deposition but also enhance the reaction kinetics of the Br_2_/Br^–^ reaction, thereby boosting the operating current density and cycle life of ZBFBs, which will unlock the potential of ZBFBs for grid-scale energy storage applications.

## 3 Conclusions

In summary, a nitrogen-doped, multiscale porous electrode has been successfully developed to simultaneously address the challenges of Zn shedding and sluggish Br_2_/Br^–^ reaction kinetics in ZBFBs. DFT calculations reveal that the incorporation of *N*-containing functional groups can boost the adsorption of Zn atoms and Br_2_ molecules to the carbon electrode, thereby achieving uniform and compact Zn deposition and fast bromine reaction kinetics. Phase field simulation demonstrates that rough electrode surfaces can enhance the mass transfer of Zn^2+^ ions, further promoting dendrite-free Zn deposition. Meanwhile, the CZGF electrodes also offer large specific surface area, providing more reaction sites for both positive and negative reactions. As a result, ZBFB using CZGF-1000 electrodes can achieve an EE of 68.0% at an ultrahigh current density of 250 mA cm^–2^, which is 17.3% higher than that of the battery equipped with PGF electrodes. Moreover, the ZBFBs exhibit excellent stability and can survive more than 800 and 2000 cycles with no obvious degradation at 250 and 100 mA cm^–2^, respectively. These results demonstrate that the newly designed electrodes can effectively address the key challenges in ZBFBs, representing a critical step toward development of high-performance ZBFBs for grid-scale energy storage.

## 4 Materials and Methods

### 4.1 Preparation of CZGF Electrodes

The GFs were purchased from the SGL carbon group, Germany. The GFs were cut into pieces of 2 × 2 cm^2^, rinsed with absolute ethanol (EtOH, 99.9%, Anaqua) and deionized (DI) water, and oven-dried at 65 °C. ZIF-8 was synthesized based on reported methods with slight adjustments.^61,62^ Specifically, 2.38 g of Zn(NO_3_)_2_·6H_2_O (99%, Aladdin) and 5.2544 g of 2-methylimidazole (98%, Aladdin) were dissolved in 40 mL of absolute methanol (MeOH, 99.8%, Anaqua) followed by ultrasonication for 30 min. The solutions were mixed, magnetically stirred for 5 min, and then immersed with GFs under continuous stirring for 12 h. The obtained GFs were thoroughly washed with EtOH. The above procedures were repeated three times. Then, the ZGFs were dried at 65 °C. The prepared ZGFs were transferred to a quartz tube furnace (OTF-1200X, Hefei Kejing) and heated to 700, 800, 900, and 1000 °C under N_2_ flow for 5 h. The ramp rate was set to 5 °C/min. Finally, the CZGFs were collected after cooling to ambient temperature.

### 4.2 Material Characterizations

The surface morphologies of different electrodes were observed by field-emission scanning electron microscopy (VEGA3). The crystal phase was analyzed via X-ray diffraction patterns obtained by an X-ray diffractometer (Rigaku SmartLab) with Cu Kβ radiation at 45 kV. The composition and chemical state of the prepared electrodes were determined by XPS (K-Alpha, Thermo Scientific). Raman spectra were collected with a WITec alpha300R Raman spectrometer. The specific surface area and pore size distribution of the samples were obtained by BET measurement (Micromeritics ASAP 2020).

### 4.3 Electrochemical Measurements

CV and EIS tests were conducted in a 0.1 M ZnBr_2_ solution with a typical three-electrode cell configuration on a BioLogic electrochemical workstation. Different GFs with a geometric area of 1 cm^2^, Ag/AgCl electrode with a salt bridge, and a Zn foil were employed as the working, reference, and counter electrode, respectively. CV tests were conducted with potential ranges of 0–1 V (vs Ag/AgCl) for the positive side and 0 to −1.2 V (vs Ag/AgCl) for the negative side at 10 mV s^–1^. The EIS test was performed from 1 × 10^5^ to 1 × 10^–1^ Hz with an amplitude of 10 mV. Charge–discharge performances of ZBFBs were assessed using a NEWARE battery test system. A Nafion 212 membrane was adopted as the separator, while 12.5 mL of solutions (1 M ZnBr_2_ + 4 M NH_4_Cl) were used as both negolyte and posolyte.

### 4.4 Computational Methods

The ABINIT code was employed for DFT calculations.^63^ Core electrons were treated with the projector augmented wave method, utilizing a plane-wave basis set with a 22 Ha energy cutoff.^64^ A 15 Å vacuum layer was applied in the slab model to avoid the influence of periodic interaction. The computational model consisted of a 4 × 4 × 1 supercell of graphite (0001) monolayer, and *K*-point Monkhorst–Pack grids of 4 × 4 × 1 were selected to sample the Brillouin zone.^65^ Geometry optimization convergence criteria were set to 4 × 10^–5^ Ha Bohr^–1^ for energy and 4 × 10^–4^ Ha Bohr^–1^ for atomic forces.

The adsorption energies (*E*_abs_) were calculated as the following formulas
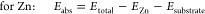

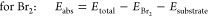
where *E*_total_, *E*_Zn_, *E*_Br_2__, and *E*_substrate_ represent the energies of the whole system, Zn atom, Br_2_ molecule and different substrate surfaces, respectively.

### 4.5 Phase Field Simulations

The dynamic morphological evolutions of Zn electrodeposition on the surfaces of both PGF and CZGF fibers are shown by phase field simulations. The modeling was based on published methods with modifications of the parameters used in the simulation.^66^ The surfaces of PGF and CZGF were constructed based on SEM morphologies. The PGF surface is smooth, while the surface of CZGF has uniform continuous protrusions. The nonconserved order parameter (ξ) tracked phase transitions, where ξ = 1 represents the solid phase, ξ = 0 corresponds to the liquid phase, and the intermediate values (0 < ξ < 1) denote the transitional interface.^67^ The relevant phase field simulation parameters for zinc deposition in ZBFB are summarized in Table S2.

## Supporting Information Available

Flow velocity distribution on the PGF and CZGF surface, and comparison of flow velocity between PGF and CZGF (Figure S1); schematic diagram of the preparation procedure of the CZGF electrodes (Figure S2); SEM images of ZIF-8 particles and ZIF-8 modified graphite felt at different magnifications (Figure S3); XRD patterns of PGF, ZIF-8 particles, ZGF, and CZGF carbonized at different temperatures (Figure S4); SEM images of CZGF-800, CZGF-900, and CZGF-1000 electrodes at different magnifications (Figure S5); full XPS spectra of CZGF-700 (Figure S6); full XPS spectra and N 1s XPS spectra of CZGF-1000 (Figure S7); pore size distribution curves of CZGF electrodes (Figure S8); CV profiles tested from 0 to −1.2 V vs Ag/AgCl and enlarged CV profiles of different samples (Figure S9); cycling performance of ZBFB using CZGF-1000 as the anode and PGF as the cathode at 100 mA cm^–2^(Figure S10); SEM images of zinc deposited on PGF and CZGF-1000 negative electrode at different magnifications after being charged at 100 mA cm^–2^ and 40 mAh cm^–2^ (Figure S11); equivalent circuits of the EIS test (Figure S12); GCD curves of ZBFBs with both PGF and CZGF-1000 (Figure S13); polarization curve and power density of the ZBFB with both PGF and CZGF-1000 electrodes (Figure S14); comparison of ZBFB performance of this work and other related works using modified electrodes (Table S1); relevant parameters of the phase field simulation model (Table S2); and abbreviations of all acronyms given in the manuscript (Table S3) (PDF)
Phase field simulation of the Zn deposition process on the PGF electrode surface (MP4)
Phase field simulation of the Zn deposition process on the designed CZGF electrode surface (MP4)
