# Supplementary material for: Reaction Kinetics and Mass Transfer Synergistically Enhanced Electrodes for High-Performance Zinc–Bromine Flow Batteries
Source: ACS Appl Mater Interfaces. 2025 Apr 18;17(17):25206–15. doi: 10.1021/acsami.4c22329 (PMC12051167; doi:10.1021/acsami.4c22329)
Supplement: Supplementary file 1 — am4c22329_si_001.pdf [file am4c22329_si_001.pdf]

Supporting information

Reaction kinetics and mass transfer synergistically enhanced electrodes for high-performance zinc-bromine flow batteries

*Jiayi Li[#], Zeyu Xu[#], Maochun Wu**

Department of Mechanical Engineering, The Hong Kong Polytechnic University, Hung Hom, Kowloon, Hong Kong SAR, China

# These authors contributed equally to this work.

* Corresponding author.

E-mail address: maochun.wu@polyu.edu.hk (M.C. Wu).

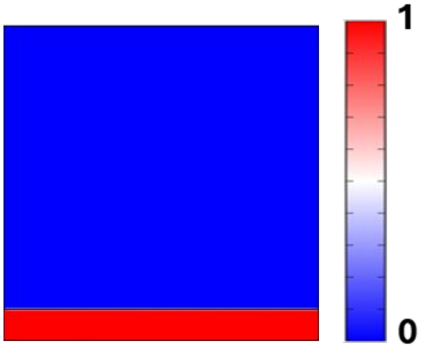

Video S1. Phase field simulation of Zn deposition process on PGF electrode surface. During the electrodeposition process, the electrode-electrolyte interface moves towards the electrolyte and becomes rough. Further growth of these uneven deposited Zn leads to the rampant formation of dendrites.

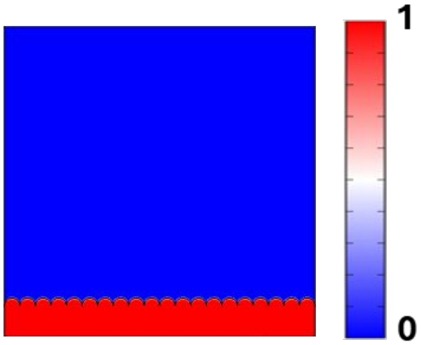

Video S2. Phase field simulation of Zn deposition process on designed CZGF electrode surface. The protrusions formed during the electrode modification process are indeed beneficial to the flat, uniform, and dendrite-free Zn formation.

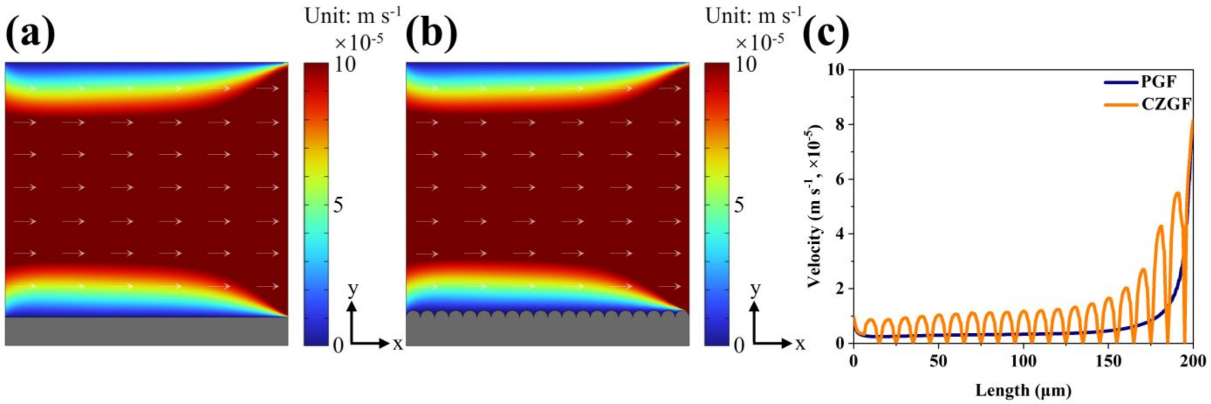

Figure S1. Flow velocity distribution on the (a) PGF surface and (b) CZGF surface. (c) Comparison of flow velocity between PGF and CZGF.

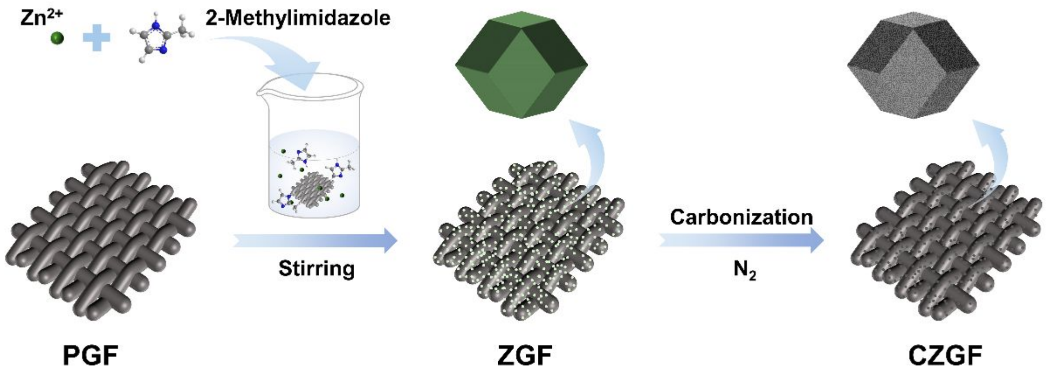

Figure S2. Schematic diagram of the preparation procedure of the CZGF electrodes.

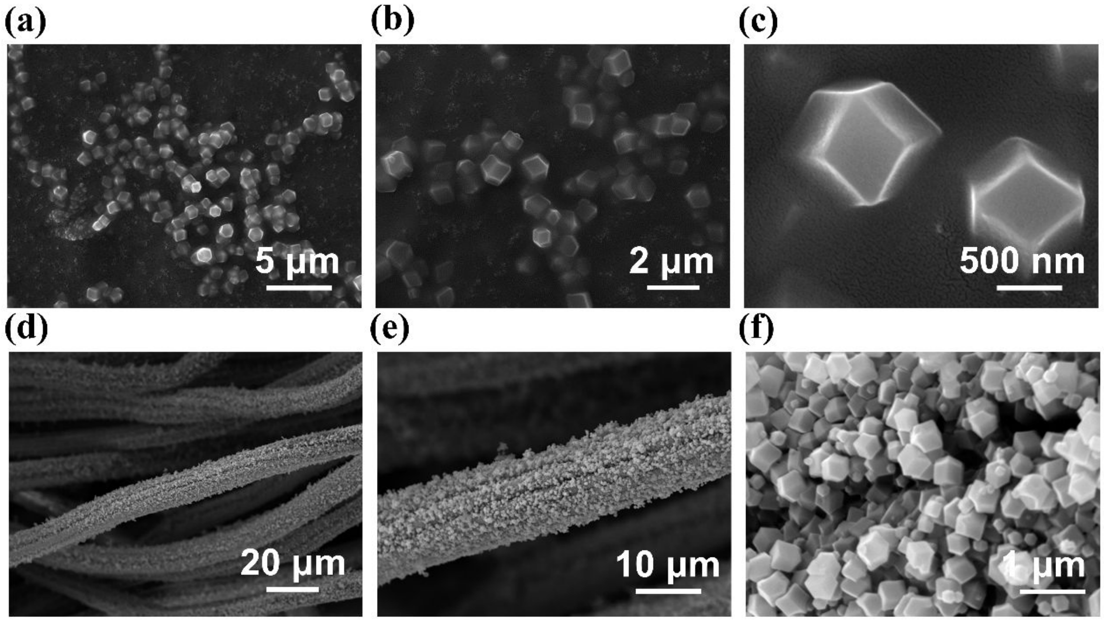

Figure S3. SEM images of (a-c) ZIF-8 particles, and (d-f) ZIF-8 modified graphite felt at different magnifications.

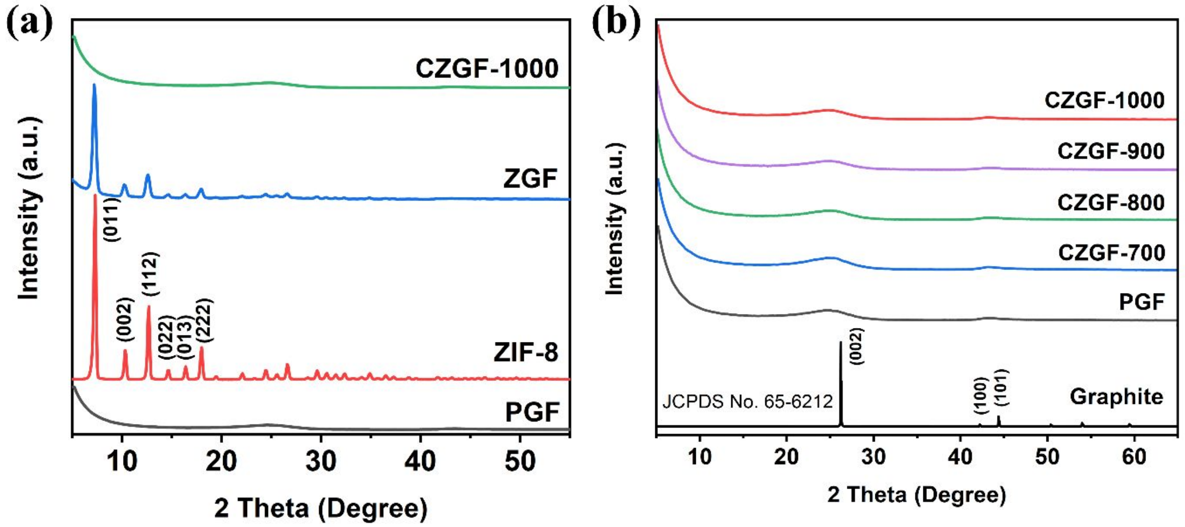

Figure S4. XRD patterns of (a) PGF, ZIF-8 particles, ZGF, and (b) CZGF carbonized at different temperatures.

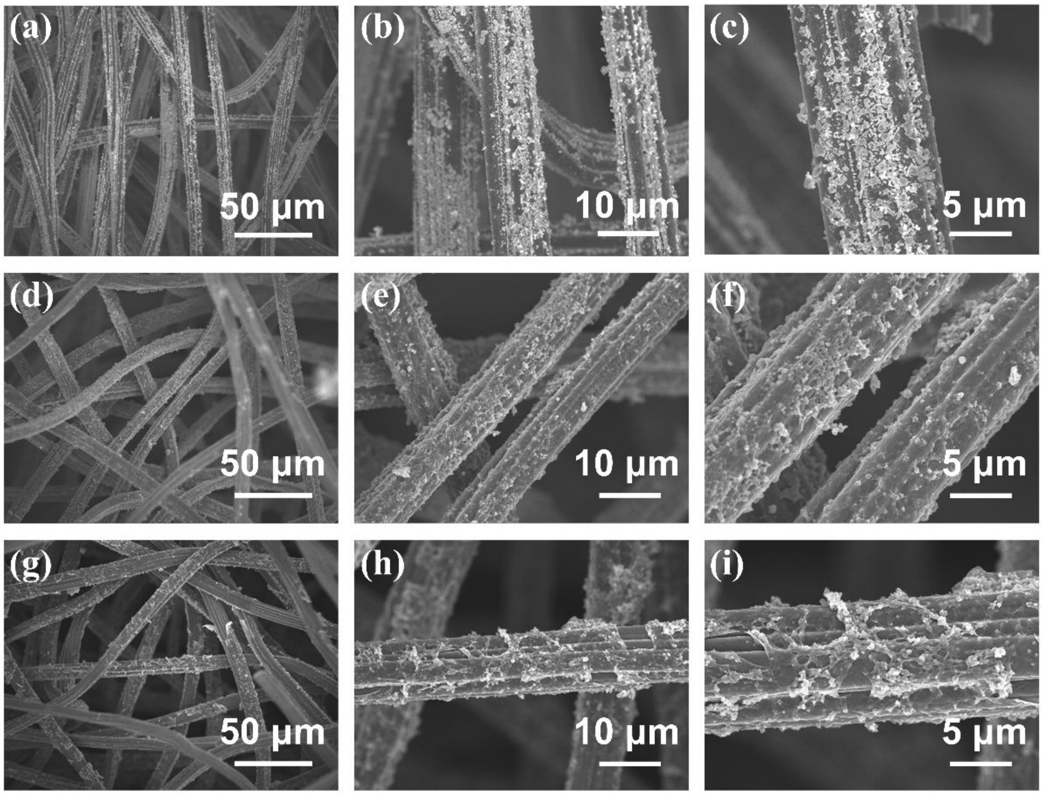

Figure S5. SEM images of (a-c) CZGF-800, (d-f) CZGF-900, and (g-i) CZGF-1000 electrodes at different magnifications.

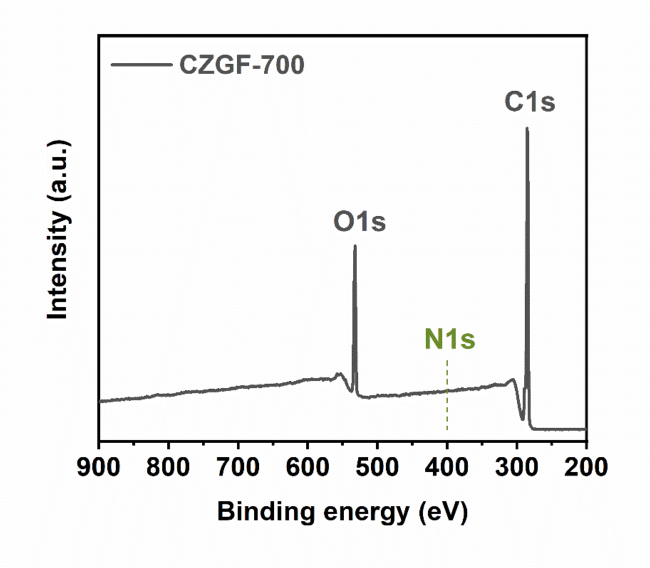

Figure S6. Full XPS spectra of CZGF-700.

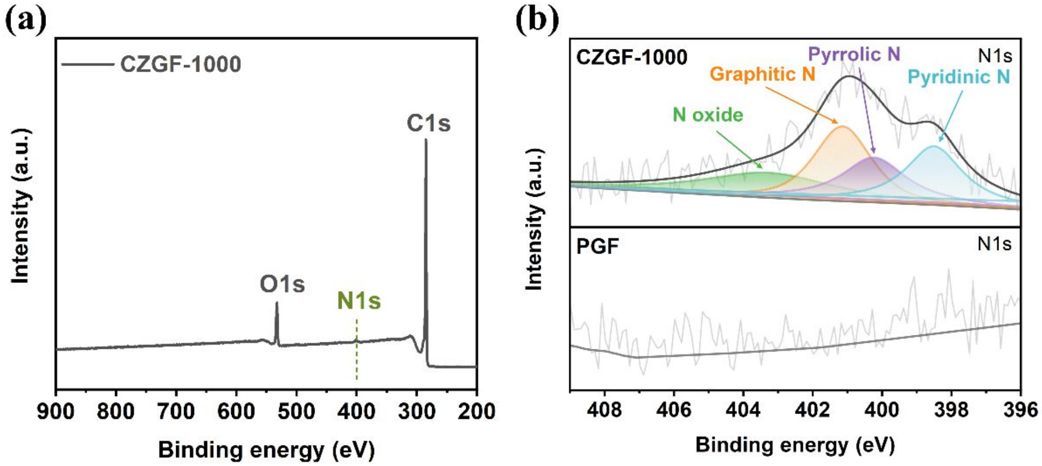

Figure S7. (a) Full XPS spectra and (b) N1s XPS spectra of CZGF-1000.

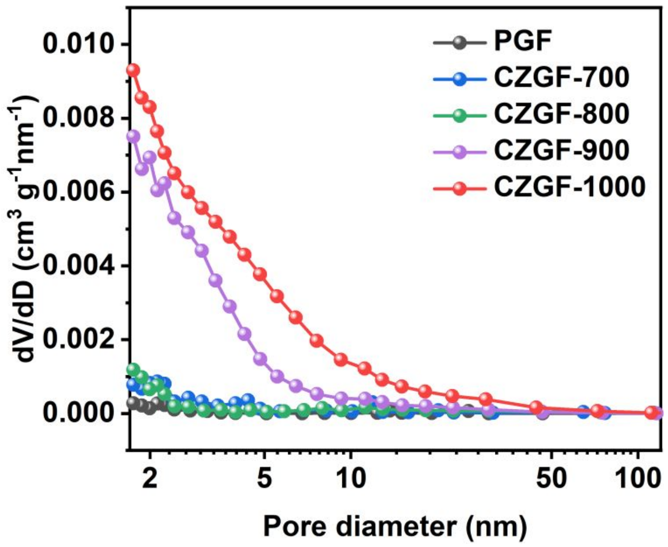

Figure S8. Pore size distribution curves of the prepared CZGF electrodes.

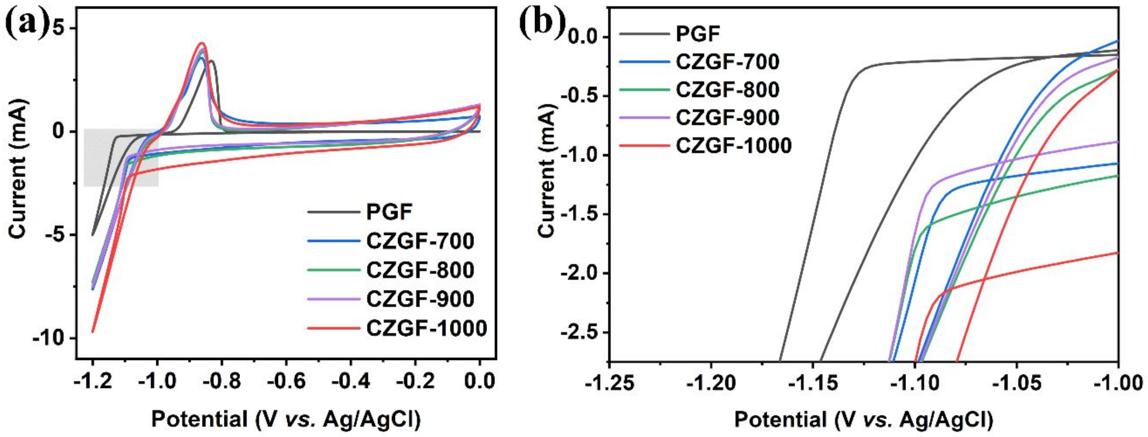

Figure S9. (a) CV profiles with the potential range from -1.2 to 0 V vs. Ag/AgCl. (b) Enlarged CV profiles of different samples.

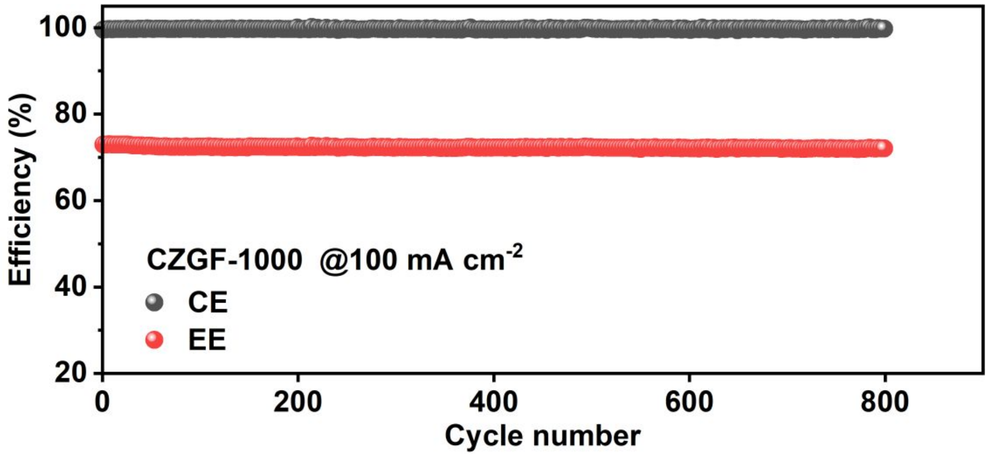

Figure S10. Cycling performance of ZBFB using CZGF-1000 as anode and PGF as cathode at 100 mA cm^{-2}.

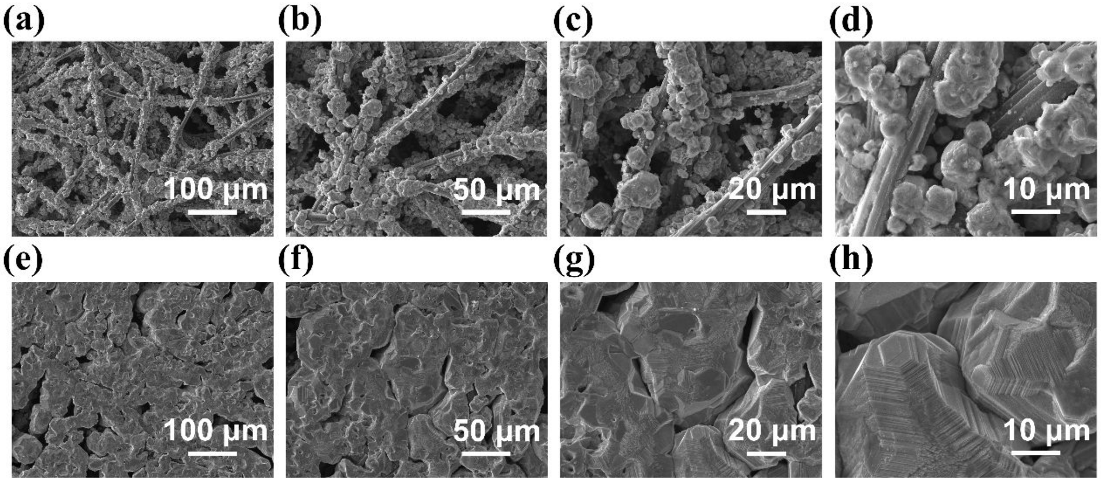

Figure S11. SEM images of zinc deposited on (a-d) PGF and (e-h) CZGF-1000 negative electrode at different magnifications after being charged at a current density of 100 mA cm^{-2} and an areal capacity of 40 mAh cm^{-2}.

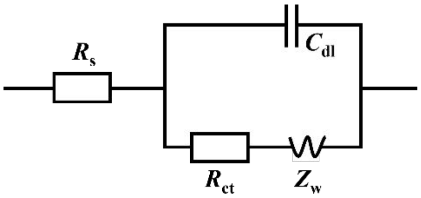

Figure S12. The equivalent circuits of the EIS test.

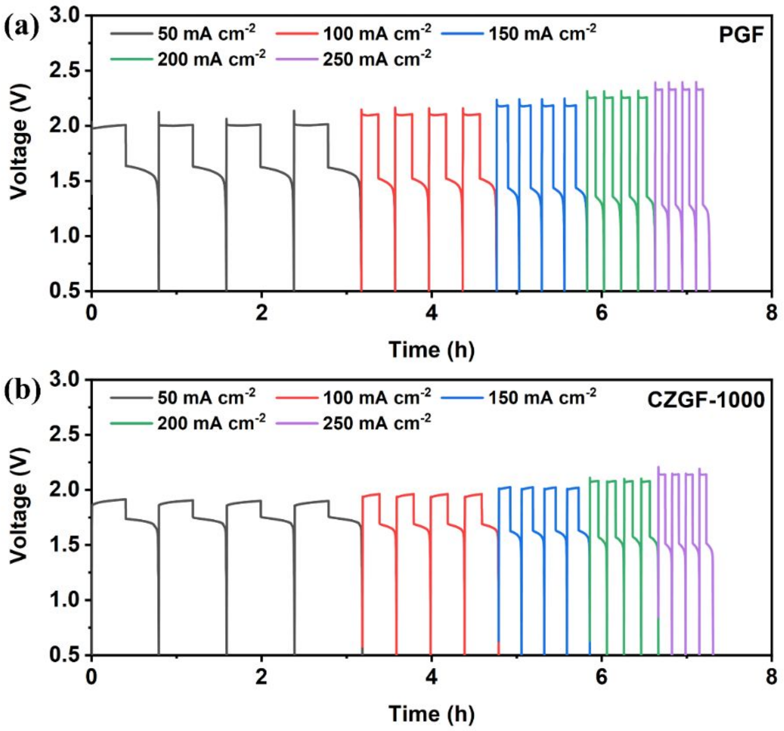

Figure S13. GCD curves of ZBFBs at different current densities with (a) PGF and (b) CZGF-1000.

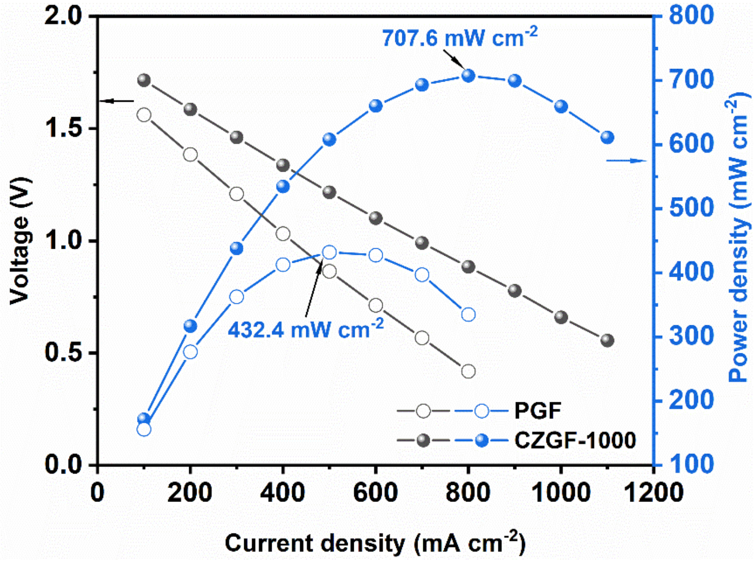

Figure S14. Polarization curve and power density of the ZBFB with both PGF and CZGF-1000 electrodes.

Table S1 Comparison of zinc bromine flow battery performance of this work and other related works using modified electrodes.

Electrodes	Current density (mA cm^{-2})	Energy efficiency (%)	Areal capacity (mAh cm^{-2})	Lifespan (cycles)	Ref.
$Pt_{0.5}Ni_1$@HT-GF	30	82.5	7.5	300	[1]
MoS_2 arrays-based CF	200	58.8	40	1000	[2]
CF-PCNS	160	~ 66	40	500	[3]
HT-N-rGO@GF	30	70.4	7.5	100	[4]
NTCF	180	~ 63.1	40	150	[5]
Bi_2O_3 felt	60	77.72	15	200	[6]
mWONNF/GF	80	80	20	400	[7]
TNHS-CF	80	82.78	40	300	[8]
R/A-TiO_2@HCS	80	83.6	40	500	[9]
PB@NC	80	85.9	40	300	[10]
CTN	160	66	40	100	[11]
**CZGF-1000**	**100**	**~ 81**	**20**	**2000**	**This work**
**CZGF-1000**	**250**	**~ 68**	**20**	**800**	**This work**

Table S2. Relevant parameters of the phase field simulation model.

Symbol	Name	Unit	Value	Refs.
L_σ	Interfacial mobility	[m^3 J^{-1} s^{-1}]	2.5×10^{-6}	[12]
ρ_e	Density of electrode	[kg m^{-3}]	7140	[13]
ρ_s	Density of electrolyte	[kg m^{-3}]	1161	[14]
α	Transfer coefficient	[1]	0.5	[15]
n	Number of electrons transferred	[1]	2	/
W	Barrier height	[J m^{-3}]	3.75×10^{5}	[16]
D_e	Diffusion coefficient in electrode	[m^2 s^{-1}]	1.0×10^{-12}	[15]
D_s	Diffusion coefficient in electrolyte	[m^2 s^{-1}]	3.0×10^{-10}	[17]
σ_e	Conductivity in electrode	[S m^{-1}]	1.7×10^{7}	[14]
σ_s	Conductivity in electrolyte	[S m^{-1}]	1	[18]

$\boldsymbol{L_\eta}$	Reaction constant	[s^{-1}]	0.001	[16]
$\boldsymbol{i_0}$	Exchange current density	[A m^{-2}]	400	[19,20]
$\boldsymbol{\kappa_0}$	Gradient energy coefficient	[J m^{-1}]	1.0×10^{-7}	[15]
$\boldsymbol{\nu}$	Electrolyte viscosity	[Pa s]	1.0×10^{-3}	[21]
$\boldsymbol{\phi_0}$	Charge voltage	[V]	0.1	/
$\boldsymbol{\omega}$	Anisotropy mode	[1]	4	[22]

Table S3 The abbreviation of all acronyms given in the manuscript.

Abbreviation	Full name
ZBFB	Zinc-bromine flow battery
GF	Graphite felt
CE	Coulombic efficiency
EE	Energy efficiency
DFT	Density functional theory
PGF	Pristine graphite felt
SEM	Scanning electron microscopy
CZGF	Carbonized ZIF-8 in situ growth on GF
ZGF	ZIF-8 modified GF
XRD	X-ray diffractometer
XPS	X-ray photoelectron spectroscope
CV	Cyclic voltammetry
EIS	Electrochemical impedance spectroscopy
R_s	Solution resistance
R_{ct}	Charge transfer resistance
$ZnBr_2$	Zinc bromide
NH_4Cl	Ammonium chloride
$Zn(NO_3)_2\cdot 6H_2O$	Zinc nitrate hexahydrate
mIM	2-Methylimidazole
EtOH	Absolute ethanol
MeOH	Absolute methanol
BET	Brunauer-Emmett-Teller
PAW	Projector augmented wave

E_{abs}	Adsorption energies
E_{total}	Energy of the whole system
E_{Zn}	Energy of the Zn atom
E_{Br2}	Energy of the Br_2 molecule
$E_{substrate}$	Energy of the substrate surface
ξ	Phase-field variable

References

(1) Mariyappan, K.; Mahalakshmi, T.; Roshni, T. S.; Ragupathy, P.; Ulaganathan, M. Nanocatalyzed PtNi Alloy Intact @3D Graphite Felt as an Effective Electrode for Super Power Redox Flow Battery. *Adv. Mater. Interfaces* **2023**, *10*, 2202007.

(2) Tang, L.; Liao, C.; Li, T.; Yuan, C.; Li, G.; Lu, W.; Li, X. In Situ Vertically Aligned MoS_2 Arrays Electrodes for Complexing Agent-Free Bromine-Based Flow Batteries with High Power Density and Long Lifespan. *Adv. Energy Mater.* **2024**, *14*, 2303282.

(3) Tang, L.; Li, T.; Lu, W.; Li, X. Lamella-like Electrode with High Br_2-Entrapping Capability and Activity Enabled by Adsorption and Spatial Confinement Effects for Bromine-Based Flow Battery. *Sci. Bull.* **2022**, *67*, 1362–1371.

(4) Mariyappan, K.; Saravanakumar, P.; Thamizhselvan, R.; Ragupathy, P.; Ulaganathan, M. In-Situ N-RGO Scaffold @3D Graphite Felt for High Power Polyhalide Hybrid Redox Flow Battery. *Adv. Mater. Technol.* **2023**, *8*, 2200869.

(5) Lu, W.; Xu, P.; Shao, S.; Li, T.; Zhang, H.; Li, X. Multifunctional Carbon Felt Electrode with N-Rich Defects Enables a Long-Cycle Zinc-Bromine Flow Battery with Ultrahigh Power Density. *Adv. Funct. Mater.* **2021**, *31*, 2102913.

(6) Naresh, R.; Velmurugan, R.; Subramanian, B.; Ragupathy, P. Laser Ablated Uniform Deposition of Bismuth Oxide Film as Efficient Anode for Zinc Based Flow Battery. *Electrochim. Acta* **2023**, *451*, 142287.

(7) Jung, H. J.; Lee, J. H.; Park, J. Y.; Shin, K.; Kim, H. T.; Cho, E. A. A Mesoporous Tungsten Oxynitride Nanofibers/Graphite Felt Composite Electrode with High Catalytic Activity for the Cathode in Zn-Br Flow Battery. *Small* **2023**, *19*, 2208280.

(8) Lai, Q.; Liu, S.; Jiang, H.; Zhang, J.; Zhou, Z.; Wang, J.; Wang, Q.; Wang, Q. Urchin-Like Mesoporous TiN Hollow Sphere Enabling Promoted Electrochemical Kinetics of Bromine-Based Flow Batteries. *Small* **2024**, *20*, 2309712.

(9) Zhang, S.; Jiang, H.; Liu, S.; Zhou, Z.; Wang, J.; Wang, Q.; Cai, K.; Lai, Q.; Wang, Q. Regulated Adsorption Capability by Interface–Electric–Field Enabling Promoted Electrochemical Kinetics of Zinc–Bromine Flow Batteries. *Chem. Eng. J.* **2024**, *486*, 150317.

(10) Zhang, Q.; Jiang, H.; Liu, S.; Wang, Q.; Wang, J.; Zhou, Z.; Cai, K.; Lai, Q.; Wang, Q. Redox-Targeting Catalyst Developing New Reaction Path for High-Power Zinc-Bromine

Flow Batteries. *J. Power Sources* **2024**, *601*, 234286.

(11) Wang, C.; Lu, W.; Lai, Q.; Xu, P.; Zhang, H.; Li, X. A TiN Nanorod Array 3D Hierarchical Composite Electrode for Ultrahigh-Power-Density Bromine-Based Flow Batteries. *Adv. Mater.* **2019**, *31*, 1904690.

(12) Wang, K.; Xiao, Y.; Pei, P.; Liu, X.; Wang, Y. A Phase-Field Model of Dendrite Growth of Electrodeposited Zinc. *J. Electrochem. Soc.* **2019**, *166*, D389–D394.

(13) Cogswell, D. A. Quantitative Phase-Field Modeling of Dendritic Electrodeposition. *Phys. Rev. E - Stat. Nonlinear, Soft Matter Phys.* **2015**, *92*, 011301.

(14) Jian, Q.; Sun, J.; Li, H.; Guo, Z.; Zhao, T. Phase-Field Modeling of Zinc Dendrites Growth in Aqueous Zinc Batteries. *Int. J. Heat Mass Transf.* **2024**, *223*, 125252.

(15) Chen, L.; Zhang, H. W.; Liang, L. Y.; Liu, Z.; Qi, Y.; Lu, P.; Chen, J.; Chen, L. Q. Modulation of Dendritic Patterns during Electrodeposition: A Nonlinear Phase-Field Model. *J. Power Sources* **2015**, *300*, 376–385.

(16) Arguello, M. E.; Gumulya, M.; Derksen, J.; Utikar, R.; Calo, V. M. Phase-Field Modeling of Planar Interface Electrodeposition in Lithium-Metal Batteries. *J. Energy Storage* **2022**, *50*, 104627.

(17) Harned, H. S.; Hudson, R. M. The Diffusion Coefficient of Zinc Sulfate in Dilute Aqueous Solution at 25°. *J. Am. Chem. Soc.* **1951**, *73*, 3781–3783.

(18) Owen, B. B.; Gurry, R. W. The Electrolytic Conductivity of Zinc Sulfate and Copper Sulfate in Water at 25°. *J. Am. Chem. Soc.* **1938**, *60*, 3074–3078.

(19) Bockris, J. O.; Nagy, Z.; Damjanovic, A. On the Deposition and Dissolution of Zinc in Alkaline Solutions. *J. Electrochem. Soc.* **1972**, *119*, 285.

(20) Jian, Q.; Wan, Y.; Sun, J.; Wu, M.; Zhao, T. A Dendrite-Free Zinc Anode for Rechargeable Aqueous Batteries. *J. Mater. Chem. A* **2020**, *8*, 20175–20184.

(21) Guerra, E.; Bestetti, M. Physicochemical Properties of $ZnSO_4$-H_2SO_4-H_2O Electrolytes of Relevance to Zinc Electrowinning. *J. Chem. Eng. Data* **2006**, *51*, 1491–1497.

(22) Kobayashi, R. Modeling and Numerical Simulations of Dendritic Crystal Growth. *Phys. D Nonlinear Phenom.* **1993**, *63*, 410–423.
